# Massively scaled variant effect mapping across the MAPK pathway reveals the importance of molecular and cellular context

**DOI:** 10.64898/2026.10.04.756099

**Published:** 2026-10-11

**Authors:** Jessica J. Simon, Sriram Pendyala, A. Felicia Adebanjo, Daniel L. Holmes, Madeline Walsh, Allyssa J. Vandi, Ziyu R. Wang, Melinda K. Wheelock, Jordan L. Opsahl, Aman Shihora, Abbye E. McEwen, Nahum T. Smith, Siobhan O’Brien, Melissa Hopkins, Alan F. Rubin, Alice H. Berger, Lea M. Starita, Dustin J. Maly, Douglas M. Fowler

## Abstract

Variation alters inherent protein properties like thermodynamic stability and catalytic activity, and these intrinsic effects are shaped by the complex cellular environment. Signaling cascades typify this complexity, with tens or hundreds of proteins acting in concert through regulated states and interactions to transduce signals. Here, we measure the effect of 186,676 variants across 17 proteins in the RAS-MAPK pathway on protein abundance and pathway activity in human cells under diverse conditions, for a total of 503,099 variant effect measurements. We discovered 14,412 dominant negative variants across 12 proteins, nearly all previously unknown, that disrupt specific domains or interaction interfaces while leaving other functions intact and that are depleted from the human population. Measuring the abundance of 106,642 variants across 10 proteins under HSP90 inhibition showed that HSP90 shapes the effect of 67% of missense variants across the pathway, buffering destabilizing variants in both strong and weak clients. Our variant effect data, collected in the context of an intact proteome, enabled us to assess the functional importance of 395 experimentally and computationally modeled MAPK protein interactions. Using additional mutational scans, we resolved how the promiscuous switch II helix binding surface of KRAS accommodates overlapping interactions with LZTR1 and SOS1, revealing distinct allosteric networks that support each interaction. Thus, we establish a framework for measuring variant effects at unprecedented scale in human cells, illuminating fundamental MAPK pathway signaling mechanisms and demonstrating that cellular context profoundly shapes the effect of most variants.

## Full Text Availability

The license terms selected by the author(s) for this preprint version do not permit archiving in PMC. The full text is available from the preprint server.

